# Factors that are associated with the risk of acquiring *Plasmodium knowlesi* malaria in Sabah, Malaysia: a case-control study protocol

**DOI:** 10.1136/bmjopen-2014-006004

**Published:** 2014-08-22

**Authors:** M J Grigg, T William, C J Drakeley, J Jelip, L von Seidlein, B E Barber, K M Fornace, N M Anstey, T W Yeo, J Cox

**Affiliations:** 1 Menzies School of Health Research and Charles Darwin University, Darwin, Northern Territory, Australia; 2 Infectious Diseases Society Sabah-Menzies School of Health Research Clinical Research Unit, Kota Kinabalu, Sabah, Malaysia; 3 Infectious Diseases Unit, Clinical Research Centre, Queen Elizabeth Hospital, Kota Kinabalu, Sabah, Malaysia; 4 Sabah Department of Health, Kota Kinabalu, Sabah, Malaysia; 5 Faculty of Infectious and Tropical Diseases, London School of Hygiene and Tropical Medicine, London, UK; 6 Royal Darwin Hospital, Darwin, Northern Territory, Australia; 7 Lee Kong Chian School of Medicine, Nanyang Technological University, Singapore

**Keywords:** PARASITOLOGY

## Abstract

**Introduction:**

*Plasmodium knowlesi* has long been present in Malaysia, and is now an emerging cause of zoonotic human malaria. Cases have been confirmed throughout South-East Asia where the ranges of its natural macaque hosts and *Anopheles leucosphyrus* group vectors overlap. The majority of cases are from Eastern Malaysia, with increasing total public health notifications despite a concurrent reduction in *Plasmodium falciparum* and *P. vivax* malaria. The public health implications are concerning given *P. knowlesi* has the highest risk of severe and fatal disease of all *Plasmodium* spp in Malaysia. Current patterns of risk and disease vary based on vector type and competence, with individual exposure risks related to forest and forest-edge activities still poorly defined. Clustering of cases has not yet been systematically evaluated despite reports of peri-domestic transmission and known vector competence for human-to-human transmission.

**Methods and analysis:**

A population-based case–control study will be conducted over a 2-year period at two adjacent districts in north-west Sabah, Malaysia. Confirmed malaria cases presenting to the district hospital sites meeting relevant inclusion criteria will be requested to enrol. Three community controls matched to the same village as the case will be selected randomly. Study procedures will include blood sampling and administration of household and individual questionnaires to evaluate potential exposure risks associated with acquisition of *P. knowlesi* malaria. Secondary outcomes will include differences in exposure variables between *P. knowlesi* and other *Plasmodium* spp, risk of severe *P. knowlesi* malaria, and evaluation of *P. knowlesi* case clustering. Primary analysis will be per protocol, with adjusted ORs for exposure risks between cases and controls calculated using conditional multiple logistic regression models.

**Ethics:**

This study has been approved by the human research ethics committees of Malaysia, the Menzies School of Health Research, Australia, and the London School of Hygiene and Tropical Medicine, UK.

**Strengths and limitations of this study**

- This study design will allow identification of *Plasmodium knowlesi* malaria risk factors, at-risk groups and case clustering, while controlling for the large number of human exposure variables related to complex monkey host and mosquito vector transmission dynamics.
- As *P. knowlesi* malaria can be acquired by all demographics, the selection of community controls allows extrapolation of exposure variables to the true population at risk, and will help to guide future public health strategies for human *P. knowlesi* malaria throughout the region.
- While potential bias in selecting community controls does exist by assuming all cases have the same access to the district hospital referral and enrolment site, this is minimised by standard Malaysian Ministry of Health malaria health policies.
- Controls matched to the same village as the case, who have lived there over the same preceding time period, will allow delineation of microepidemiological risk exposures on an individual and household level and will also assist in minimising bias related to differences in case accessibility to the enrolment sites.
- Not matching controls to the case's age and gender gives less power to detect differences in other individual exposure risks within these demographics; however, it does allow analysis of these as potential independent risk factors, including their relationship with disease severity.

## Introduction

### *Plasmodium knowlesi* as an emerging public health problem in Malaysia

The first naturally acquired human infection of the simian malaria parasite *Plasmodium knowlesi* was reported in 1965 in Pahang, Peninsular Malaysia.^1^ A survey of 1117 people living in the vicinity of the case failed to produce further evidence of *P. knowlesi* human infection.^2^ Subsequent screening for *P. knowlesi* was limited due to the assumed scarcity of zoonotic human malaria and an inability to differentiate morphologically between *P. knowlesi* and *P. malariae* using routine microscopy.^3^ ^4^ In 2004, atypical *P. malariae* microscopy reported cases were correctly identified by PCR as a substantial focus of human *P. knowlesi* infections at Kapit in Sarawak, Eastern Malaysia.^5^ *P. knowlesi* is also commonly misreported by microscopy as either *P. falciparum* or *P. vivax* in endemic areas such as Sabah, Malaysia,^4^ ^6^ where state guidelines currently only require routine referral of microscopic *P. malariae* cases for confirmatory PCR speciation.^7^

Current evidence suggests that *P. knowlesi* has long been present in Eastern Malaysia.^6^ ^8^ ^9^ Analysis of *P. knowlesi* mtDNA sequences from Sarawak suggest that *P. knowlesi* was present in local macaques before the arrival of humans in South-East Asia^10^ and a large increase in the parasite population corresponds to the initial rise in the human population in this region around 30 000–40 000 years ago.^11^ The earliest PCR confirmation of *P. knowlesi* comes from archival slides from Sarawak (originally diagnosed as *P. malariae*) dating back to 1996.^12^ Recent studies in Eastern Malaysia have consistently shown that only a very small proportion (<1%) of *P. malariae* cases diagnosed by microscopy can be confirmed by PCR. The vast majority of these cases are, in fact, *P. knowlesi*.^5^ ^8^ ^13–17^ By extension it is possible that a significant proportion of *P. malariae* cases reported historically in Eastern Malaysia were, in reality, *P. knowlesi* infections. The earliest malaria surveys conducted in Tambunan, Sabah, between 1939 and 1942 described high-intensity malaria foci in hilly ravines with unconfirmed *P. malariae*.^18^ In Sarawak, results from the first recorded microscopy-based surveys conducted in 1952 indicated that *P. malariae* accounted for 33% of all malaria cases and up to 76% in some areas.^2^

In Sabah, recently, a marked, rapid increase in the number of *P. knowlesi* cases beginning in the south-west and progressing north-easterly has been reported.^19^ Public health microscopy notifications for *P. knowlesi* in Sabah (including those reported as *P. malariae*) have increased from around 2% (59/2741) of all malaria cases in 2004, to 35% (703/1936) in 2011^19^ and, recently, to 62% in 2013 (996/1606) (Sabah Department of Health, unpublished). The rise in *P. knowlesi* notifications in Sabah may also be underestimated given PCR-confirmed *P. knowlesi* is misdiagnosed as *P. falciparum* or *P. vivax* in up to 13% and 10% of cases, respectively, by routine microscopic examination in this area.^4^ In Sarawak, government health reports of *P. knowlesi* or *P. malariae* microscopy notifications totalled 14.3% (1731/12 082) of all malaria cases between 2000 and 2006,^9^ increasing to 41% (897/2189) in 2009^15^ and to 73% (737/1004) in 2013.^20^ The change in *Plasmodium* spp proportions in both Sabah and Sarawak coincides with an effective national Malaria Control Program implemented in Eastern Malaysia in 1971,^21^ with a consequent decrease in *P. falciparum* and *P. vivax* cases.^19^ However, this does not explain the differences in transmission dynamics that have resulted in a marked increase in absolute numbers of *P. knowlesi* cases in Sabah but not in Sarawak.

Since 2004, sporadic cases of PCR-confirmed *P. knowlesi* human malaria have also been reported in Peninsular Malaysia and a number of Asian countries including Thailand, Vietnam, Cambodia, Myanmar, Singapore, Brunei, Indonesia, India, China and the Philippines.^8^ ^22–30^ Recent risk maps suggest that large areas in this region have the necessary pre-requisites for an infectious *P. knowlesi* reservoir^31^ and as molecular diagnosis is not routine outside Malaysia, it is possible that a substantial number of *P. knowlesi* infections are going undetected. However, existing evidence also suggests that *P. knowlesi* incidence is relatively low in areas where other human *Plasmodium* spp predominate.^23^ ^32^ Outside Malaysia, *P. knowlesi* occurs most commonly within mixed infections^23^ ^27^ ^33^ and has not yet been shown to cause severe disease. In Malaysia, by contrast, *P. knowlesi* is associated with a threefold risk of severe disease compared with *P. falciparum*^17^ and fatal outcomes have been reported.^34^ ^35^

### Current understanding on epidemiological patterns of *P. knowlesi* risk

Currently it is unclear why patterns of *P. knowlesi* infection and disease in at-risk populations vary so markedly across this region. Overall, the distribution of cases is constrained by the ranges of the predominant natural hosts for *P. knowlesi*: long-tailed (*Macaca fascicularis*) and pig-tailed (*M. nemestrina*) macaques.^36^ Transmission also depends on the presence of competent mosquito vectors within the *Anopheles leucosphyrus* group,^37^ which, together with factors that determine the nature of vector-human contact, results in a spatially heterogeneous pattern of *P. knowlesi* transmission and more fundamental variations in the epidemiology of the disease. In Kapit, Sarawak, for example, *A. leucosphyrus* (previously described as *A. latens*) was identified in 2006 as a *P. knowlesi* vector of both humans and monkeys and demonstrated relatively high human biting rates within a farming area at the forest fringe.^38^ These entomological findings were consistent with results from an epidemiological study carried out at Kapit Hospital in 2006–2008, during which 87% (93/107) of patients who were prospectively admitted with *P. knowlesi* malaria reported having engaged in recent undefined forest or forest-edge activities.^39^ The epidemiological picture in Sabah, where *Anopheles balabacensis* (the vector used in human *P. knowlesi* transmission studies in the 1960s^40^) is assumed to be the main vector for *P. knowlesi*,^41^ ^42^ is hypothesised to be different. However, a prospective study of malaria patients from a referral catchment area of north-western Sabah also described 92% (119/130) of PCR confirmed *P. knowlesi* malaria cases having forest or plantation exposure. Despite this, patients with *P. knowlesi* in this study were no more likely than patients with *P. falciparum* to report staying overnight or spending >4 h in a forest or plantation in the previous 1 month.^17^ In contrast, living within a 20 min walk of unclassified forest type or a plantation did appear related (p=0.001 and 0.015, respectively), in addition to a positive history of sighting a monkey in the previous 4 weeks (p<0.001).^17^

While comparisons in *P. knowlesi* transmission patterns between Sabah and Sarawak remain difficult given broad definitions of forest and forest-edge exposure in previous studies, differences in more specific related factors may be impacting on the patterns of clustering of *P. knowlesi* cases. Initial studies from Sarawak had shown a higher prevalence of *P. knowlesi* malaria in adult males with occupational forest exposure, with no clustering of cases despite the fact that most people live in communal longhouses.^5^ Data from Sabah have also documented up to 78% of *P. knowlesi* cases are male^17^ ^19^ while for female cases a bimodal age distribution was described, which may still indicate that risk of infection is predominantly linked to exposure activities.^19^ In addition, no relationship with occupation was seen when comparing *P. knowlesi* with *P. falciparum* cases.^17^ ^19^ However, a study at Kudat at the north-western tip of Sabah showed clear differences to Sarawak with both a wide age distribution of cases and contemporaneous family clusters including children and town residents, suggesting peridomestic transmission is also occurring.^43^ *P. knowlesi* cases in Sabah have also been shown to demonstrate temporal clustering, with marked seasonality (p<0.001) associated with a peak of cases in May and 3–5 months post-high rainfall.^19^ ^43^

Evidence of peridomestic transmission in Sabah raises the possibility of human-to-human transmission. To date, this is not evident from focused studies outside Sabah where macaque and human samples have been compared. These include the analysis of *P. knowlesi* CSP or MSP gene sequences in Sarawak and Peninsular Malaysia, and also in other countries including Singapore and Thailand, which all showed a large number of shared haplotypes that were similarly diverse in both hosts, supporting a zoonotic rather than exclusively human transmission mode.^10^ ^32^ ^44^ ^45^ In addition, no evidence of ongoing human *P. knowlesi* transmission in areas without a macaque reservoir has been documented. However, in the absence of large-scale molecular studies comparing humans and macaques in this region, and given human-to-human transmission has been demonstrated under experimental conditions,^40^ the occurrence of natural human-to-human *P. knowlesi* transmission alongside monkey-to-human transmission remains possible.

### Key knowledge gaps in epidemiological determinants of *P. knowlesi* risk

*P. knowlesi* acquisition risk factors related to behaviour or environment currently remain poorly defined even in the more intensively studied areas of Eastern Malaysia. Changes in land use such as shifts in agricultural practices, deforestation and forest fragmentation have been proposed as key drivers in the emergence of *P. knowlesi* infection.^8^ However, there has been no detailed evaluation of potential risk factors related to household characteristics or local patterns of land use/land cover. In addition, there is limited understanding of human movement patterns or travel history related to differences in forest or forest edge exposure, including duration, frequency and timing of activities. Occupational exposure has not been evaluated in a systematic manner, either with respect to seasonal practices or differences associated with rubber or palm oil plantations versus traditional freeholder farming. To date, no studies have looked in detail at human and monkey interaction, considering the zoonotic transmission mode of *P. knowlesi* and the potential novel risks related to encroachment of humans on macaque habitats and vice versa. This interaction could relate to proximity of vectors and include conflict around availability of food for macaques from human agricultural practices such as cultivated fruit orchards, rice paddies or cornfields, in addition to traditional or changing practices such as trapping or hunting monkeys for pets or food.

Current reported studies have not been designed to control for other human behavioural or immunological factors such as the documented higher incidence of *P. knowlesi* malaria in older age^17^ ^39^ ^43^ ^46^ or the risk of *P. knowlesi* malaria during pregnancy. Age as a demographic factor alone also needs investigation, given widespread reports of *P. knowlesi* cases in children in Malaysia and elsewhere in South-East Asia.^23^ ^46^ ^47^ In addition, no studies have evaluated whether genetic factors affect individual *P. knowlesi* malaria acquisition risk, such as seen with the protective nature of certain human red blood cell polymorphisms against other *Plasmodium* spp.^48–50^

### Case–control study designs to identify risk factors for malaria

Case–control study designs have been used in areas of low endemicity in the past to identify risk factors for malaria due to other *Plasmodium* spp.^51^ Existing efforts to fill knowledge gaps in individual risk for human *P. knowlesi* malaria focus on delineating activities at the forest edge and within the forest, and differentiating these in terms of occupational risk and those related to domestic behaviour. As the current incidence of *P. knowlesi* malaria in Sabah is relatively low, a prospective cohort study design would not be an efficient way of assessing these relative risks because of the very large number of participants that would need to be recruited. In addition, with a cohort study the loss to follow-up of any participants would negatively impact the causal analysis of the primary outcome of interest, which is the acquisition of *P. knowlesi* malaria. The case–control design is also particularly well suited in identifying at-risk groups or clustering at a finer epidemiological scale as it allows controlling for the complex and large number of human exposure variables involved in the transmission dynamics of *P. knowlesi* infection. In addition, this design is useful for generating hypotheses on *P. knowlesi* acquisition that can be tested using other future study designs such as cross-sectional surveys, which are more suited to providing detailed information on population-wide *P. knowlesi* prevalence including asymptomatic infection.

## Aim and objectives

The study design described in this protocol will allow evaluation of the primary research question: “Which factors lead to increased risk of acquiring human *P. knowlesi* malaria in Sabah, Malaysia?” Secondary objectives will include comparisons of malaria acquisition risk between different *Plasmodium* spp, risk of severe disease within the *P. knowlesi* group of malaria cases, and evaluation of *P. knowlesi* clustering.

Information resulting from this study will assist in guiding public health strategies for malaria in this region and provide valuable human epidemiological data for integration with concurrent studies on the entomology, primatology and land use aspects of *P. knowlesi* transmission (Monkeybar project: UK MRC ESEI Grant #G1100796) and the clinical treatment of *P. knowlesi* malaria (clinicaltrials.gov #NCT01708876).

## Methods and analysis

*Current status*: Enrolment started in December 2012, with 189 *P. knowlesi* cases and 564 community controls as on June 2014.

### Study design

**Figure 1:**
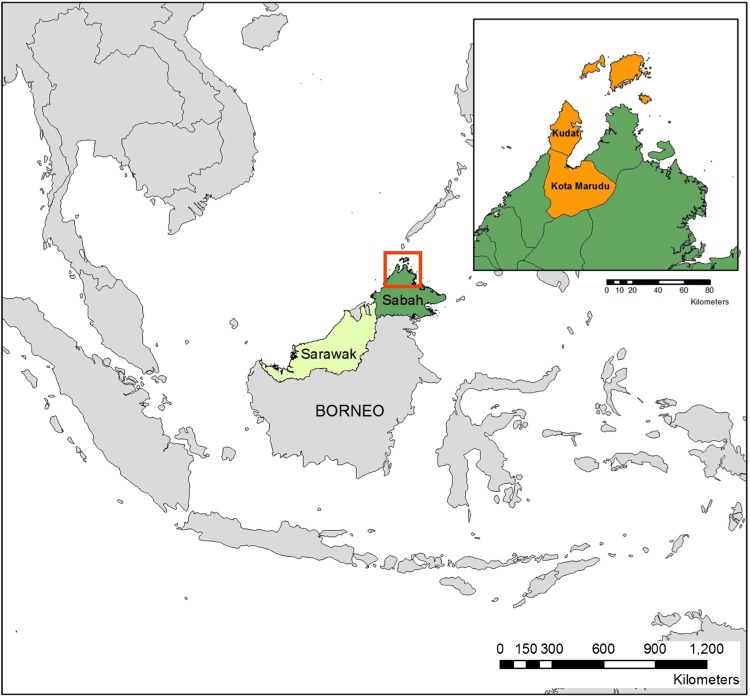
Map of study sites.

This will be a population-based case–control study conducted in two adjacent districts: Kudat and Kota Marudu in the state of Sabah, Malaysian Borneo (figure 1). Within the study sites, all malaria cases, regardless of parasite species, meeting specified inclusion criteria will be enrolled at the corresponding district referral hospital over a 2-year period. Research fieldworkers will go to the village of the case within 2 weeks of focal case detection and randomly select three community controls who have primarily resided in the village for the past 3 weeks. This will enable matching of the controls with the case by the same geographical area (local village) and preceding minimum time period of exposure risk (3 weeks).

#### Study sites

Kudat and Kota Murudu districts cover an area of 3204 km^2^ over the north-western tip of the state of Sabah, Malaysian Borneo and have a combined population of around 150 000 (2010 data^52^). These sites were chosen predominantly because of documented high incidence of *P. knowlesi* infection; in particular with *P. knowlesi* being the predominant species in Kudat.^43^ ^46^ Kudat and Kota Murudu districts have central referral hospitals serving defined catchment areas with large outpatient and inpatient facilities. Recruitment was also expanded to include patients presenting primarily to Kota Marudu District Hospital from the neighbouring Pitas district, with a higher proportion of cases due to other *Plasmodium* spp enabling secondary objectives to be evaluated.

#### Community controls

Because *P. knowlesi* can be acquired by either sex and at any age,^34^ ^39^ ^43^ ^46^ community controls can provide an accurate representation of the study base population. This enables extrapolation of explored variables to the true population at risk.^53^ Choosing controls with no history of fever minimises possible confounding from any concurrent acute illness. By not choosing hospital controls there is some bias generated in assuming controls enrolled out in the community have the same access to the hospital study site as cases. The choice of community controls may also create bias by assuming that the malaria cases that present to the hospital and are enrolled genuinely account for all malaria cases in the community, that is, the study base population. However, in the current setting, factors that increase the likelihood of ascertaining all cases include (1) mandatory state reporting and hospital referral of malaria cases from government and private clinics; (2) the presence of only a single district referral hospital in each of the two study sites and (3) the provision of free or relatively inexpensive treatment at government health facilities. State Ministry of Health guidelines also dictate that a blood film for malaria should be prepared routinely for any patient who presents to a government health facility with fever.

#### Matching controls to the case's home village

This further minimises case ascertainment bias related to variations in geographical accessibility to the main referral study site. Matched village controls may reside anywhere within the study districts, whereas hospital controls may potentially come from a more restricted geographical area around the hospital. This design also provides greater statistical power to evaluate microepidemiological features related to individual and household factors. This was deemed more important than a design using hospital controls, which would have enabled comparisons between different locations and villages, but which would have had less power to delineate individual behaviour and demographic exposure risks.

#### Matching controls for preceding time exposure

Controls will be enrolled within a 2-week time period following identification of the case. Only individuals who have resided in the study area over a 3-week period prior to the presentation of the matched case will be eligible. This will encompass the prepatent time period of *P. knowlesi* infection of around 9–12 days.^40^ Analysis of potential relationships that vary over time such as seasonal planting or harvesting, other agricultural practices, cultural events or meteorological data will be possible.

#### Non-matching controls for age/gender

While this would have given additional power to detect differences in exposure risks within these demographics, it was decided that age and gender should be evaluated as potential independent risk factors. This will also allow comparisons between *Plasmodium* spp and with disease severity related to age or gender.

#### Recruitment/sample size

We aim to recruit all cases of confirmed malaria of any species or severity over the study duration from Kudat and Kota Murudu district hospitals. On the basis of current recruitment rates of around 10 malaria cases per month over the last year from both sites, we expect to enrol approximately 240 cases of *P. knowlesi* malaria and an additional 90 cases of malaria due to other species in the period December 2012 to December 2014. For the 330 malaria cases we will recruit 990 matched controls. A recruitment logbook will be kept for all screened cases and controls to document reasons for non-eligibility, which will be included in the analysis. With three controls per case, assuming probability of exposure among controls (Po) is 0.1, and the relative risk (RR) of acquiring *P. knowlesi* malaria in exposed subjects relative to unexposed subjects is 0.3, we will need to study a minimum of 210 *P. knowlesi* cases to be able to reject the null hypothesis that there is no difference between cases and controls (ie, relative risk=1), with 80% power and an α level of 0.05.^54^

#### Definition and selection of cases

All male or female patients of any age presenting to a study site with confirmed malaria.

*Inclusion criteria:* Positive microscopy for *Plasmodium* infection; documented fever or history of fever in the past 48 h; primarily resided in the study area in the previous 3 weeks; residence can be located and mapped; appropriate informed consent obtained.

*Exclusion criteria:* Previously enrolled as a case (repeat malaria infection); age <4 weeks; subsequent PCR negative for *Plasmodium* spp monoinfection.

Cases will be classified according to PCR confirmation of *Plasmodium* spp monoinfection. Severe and non-severe *P. knowlesi* cases will be defined by whether they meet modified WHO 2010 severe malaria clinical and laboratory criteria as described elsewhere.^17^

#### Definition and selection of community controls

A systematic selection procedure will be used to recruit three community controls for each case matched to the same village. This number of controls was chosen to provide sufficient statistical power to measure exposure effects, while at the same time making the recruitment process feasible logistically. Selection of control households and individuals within these households will be conducted using a validated and pretested random method based on up-to-date Malaysian Public Health Department village maps and household lists. Corroboration of the number of households will be undertaken with local residents, public health officers and village heads, where available. Households will be numbered using either the map and/or the household head of family name. A smartphone application generates a random number corresponding to one of the households. Individuals primarily residing within the selected household in the previous 3 weeks will be numbered and then randomised by the same application. All households within the village and all individuals within that household will have an equal chance of being selected. If the selected individual is not present when the fieldworkers visit the household, then they will return up to three times, which may include an arranged later time on the same day or on subsequent days. This will minimise selection bias related to the higher likelihood of male adults or school age children not being present at the household during the day. If it is not possible to obtain informed consent from a selected individual or if the specified inclusion criteria are not met, another individual within the same house will be selected based on the next randomly generated number. Failing this an alternative household will be selected using the same method.

*Control inclusion criteria:* Appropriate informed consent obtained; agree to finger-prick blood sample; negative blood slide and PCR result for malaria (delayed); primarily resided in the study area in preceding 3 weeks; residence can be located and mapped; no history of fever in previous 48 h.

*Control exclusion criteria:* Previously recruited as a case; previously recruited as a control; member of a previously selected control household; age<4 weeks.

### Study procedures

#### Follow-up

**Figure 2:**
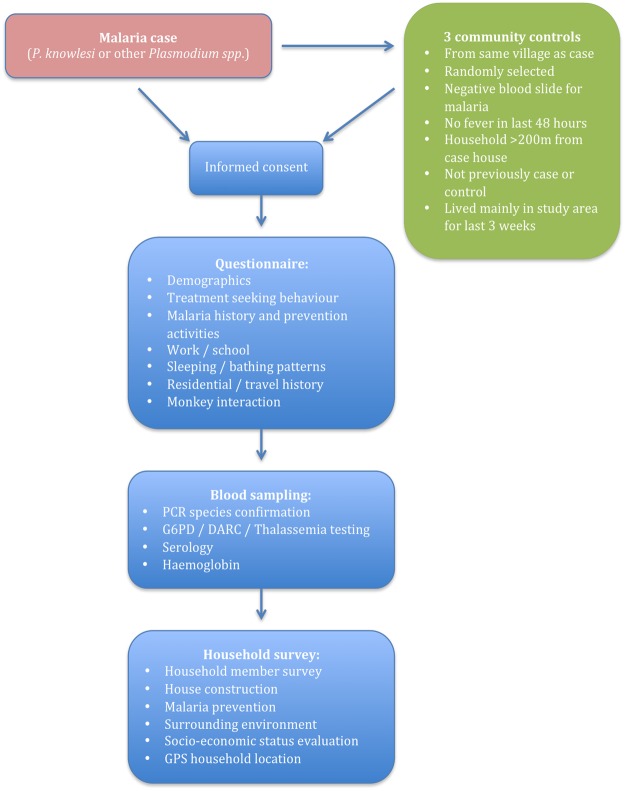
Enrolment/study procedures flow chart.

Both cases and controls will require a single visit to their homes to administer the questionnaire and household survey and to carry out blood sampling and GPS mapping. This will occur within a 2-week period after the index case detection (see figure 2).

#### Questionnaire

A standardised pretested questionnaire was developed with input from multiple relevant disciplines including public health, epidemiology, clinical, entomology, primatology and social science. The questionnaire will be administered to cases and controls by trained fieldworkers who are native speakers in Bahasa Malaysia or other local dialects. The questions will elicit possible associations of malaria with several categories of potential environmental, socioeconomic and clinical risk factors, particularly in the context of potential interaction with primate hosts and vectors. Owing to the nature of case and control recruitment it will not be possible to blind fieldworkers to the participant's recent malaria status. For children or younger participants who are either not able or are uncomfortable answering the questions, the household head or parent/guardian will be asked to answer from the participant's perspective. Participants who do not speak Bahasa Malaysia or other local dialects, or who are unable to answer the questions for other reasons, will have an appropriate family member answer for them.

#### Household survey

Fieldworkers will conduct a household survey to record data for a range of variables including household construction and utilities, malaria prevention methods used, ownership of assets, characteristics of the surrounding environment and other factors relating to potential monkey and vector exposure. All households will be mapped using a GPS receiver.

#### Economic status

An index of socioeconomic status will be derived using established methods.^55^ ^56^ The index will incorporate locally validated surrogate markers of income including access to services (electricity, water, sanitation) and ownership of specific assets (motorbike, car, refrigerator, generator or television).

#### Blood sampling

Blood collection will be requested at time of enrolment for cases, controls and additional members of control households. A venepuncture or finger-prick will be performed for cases and members of control households, respectively. No additional blood will be collected from cases already enrolled in parallel clinical studies (which include all assays required for the case–control study). Whole blood will be used for preparation of Giemsa stained thin and thick blood films to assess for malarial parasites, and also collected using 3 ×20 µL spots on filter paper (3MM; Whatman, UK) and a 500 µL EDTA tube (Becton-Dickinson, New Jersey, USA). PCR will be performed using previously described methods for *P. knowlesi*^57^ and *P. falciparum*, *P. vivax*, *P. ovale* and *P. malariae*^58^ detection. Haemoglobin will be assessed using a portable spectrophotometer (HemoCue AB, Angelholm, Sweden) for controls or using standard hospital laboratory methods for cases. Testing for red blood cell polymorphisms potentially associated with *P. knowlesi* acquisition will be performed including glucose-6-phosphate dehydrogenase (G6PD) deficiency, Duffy antigen receptor for chemokines (DARC) and α and β thalaessemia. Blood on filter paper spots will be stored for potential future serological analysis of historical changes in exposure to *P. knowlesi* infection, as has previously been done for other *Plasmodium* spp.^59^

### Statistical plan

#### Primary

The exposures of interest to be compared between *P. knowlesi* malaria cases (PCR-confirmed monoinfection) and controls will include variables relating to demographic, behavioural and socioeconomic factors, red blood cell polymorphisms and serological and immunological blood markers. Analysis will be per protocol, with ORs (including 95% CIs) for exposure risk between cases with *P. knowlesi* infection and controls calculated by the Mantel-Haenszel method and then compared. Confounding variables will be adjusted for using conditional multiple logistic regression models. Normally and non-normally distributed numerical variables will be analysed using t tests and Mann-Whitney U tests, respectively. An initial analysis will be conducted for cases and matched controls with complete variable data sets. The extent of missing data will be assessed and the sensitivity of results to alternative approaches to imputing missing data will be explored, if considered necessary.

#### Secondary

A similar analytical approach to the above will be used. Exposures of interest will be compared between all *P. knowlesi* and other *Plasmodium* spp malaria cases, as well as between severe and non-severe *P. knowlesi* malaria. Temporal and spatial patterns of molecularly confirmed cases will be described and potential correlations with meteorological and other environmental variables will be explored using data from automatic weather stations and remote sensing satellites. PCR assessment of prevalence of asymptomatic parasitaemia of any species, pregnancy outcomes and burden of anaemia associated with different malarial parasite species will also be detailed.

#### Exclusion/censoring

Any cases found to have subsequent negative PCR for *Plasmodium* spp will be excluded in the analysis, while those with mixed *Plasmodium* spp infection will be censored in the analysis. Any controls who are subsequently shown to have a positive blood film or PCR result for any *Plasmodium* spp at the time of enrolment will be censored in the analysis. Data from participants withdrawing consent will be censored in the analysis after that time-point. For controls who withdraw, replacements will be sought as long as it is still possible to meet inclusion criteria.

## Ethics and dissemination

### Ethics

The study will adhere to the principles that govern biomedical research involving human subjects. The Declaration of Helsinki will be followed to provide assurance that the rights, integrity and confidentiality of study subjects are protected and that reported results are credible and accurate. Written informed consent will be obtained from participants or parents/guardians if <18 years of age. Any potential controls or household members found to have a positive screen for malaria will be treated as per standard Malaysian Ministry of Health guidelines. No payments will be made to participants. This study has been approved by the human research ethics committees of Malaysia (MREC), the Menzies School of Health Research, Australia and the London School of Hygiene and Tropical Medicine, UK.

### Dissemination of results

Plain language summaries translated into Bahasa Malaysia will be distributed through subsector public health clinics. Community meetings will be held in the study districts to inform community members/participants. Relevant state and national Malaysian Ministry of Health bodies will be provided with reports and publications in a prompt manner.
